# The genome sequence of the Large Skipper, *Ochlodes sylvanus*, (Esper, 1777)

**DOI:** 10.12688/wellcomeopenres.18788.1

**Published:** 2023-02-14

**Authors:** Konrad Lohse, Alex Hayward, Roger Vila, Wellcome Sanger Institute Tree of Life programme, Wellcome Sanger Institute Scientific Operations: DNA Pipelines collective, Tree of Life Core Informatics collective, Ana Paula S. Carvalho, Akito Y. Kawahara, Darwin Tree of Life Consortium

**Affiliations:** 1 Institute of Ecology and Evolution, University of Edinburgh, Edinburgh, UK; 2 College of Life and Environmental Sciences, Department of Biosciences, University of Exeter, Exeter, UK; 3 Institut de Biologia Evolutiva (CSIC - Universitat Pompeu Fabra), Barcelona, Spain; 4 McGuire Center for Lepidoptera & Biodiversity, University of Florida, Gainesville, Florida, USA

**Keywords:** Ochlodes sylvanus, Large Skipper, genome sequence, chromosomal, Lepidoptera

## Abstract

We present a genome assembly from an individual female *Ochlodes sylvanus*, the Large Skipper (Arthropoda; Insecta; Lepidoptera; Hesperiidae). The genome sequence is 380 megabases in span. Most of the assembly (99.97%) is scaffolded into 30 chromosomal pseudomolecules, including the assembled W and Z sex chromosomes. The mitochondrial genome has also been assembled and is 17.1 kilobases in length. Gene annotation of this assembly on Ensembl identified 13,451 protein coding genes.

## Species taxonomy

Eukaryota; Metazoa; Ecdysozoa; Arthropoda; Hexapoda; Insecta; Pterygota; Neoptera; Endopterygota; Lepidoptera; Glossata; Ditrysia; Hesperioidea; Hesperiidae; Hesperiinae; Hesperiini; *Ochlodes*; *Ochlodes sylvanus* (Esper, 1777) (NCBI:txid876063).

## Background

The Large Skipper, *Ochlodes sylvanus*, is a Palearctic butterfly in the family Hesperiidae, found from western Europe to eastern Russia and Japan. The species used to also be referred to as *O. venatus*, which however is now generally considered a similar species occurring in East Asia ( Devyatkin, 1997). In the British Isles, the Large Skipper is restricted to England and Wales and southern parts of Scotland (and absent from Ireland). The species is currently undergoing a northwards range expansion in the UK, most likely in response to climate change.

The Large Skipper is one of the most common skippers in Europe and a species of Least Concern both on the IUCN Red List of Europe ( van Swaay *et al.*, 2010) and the revised Red List for the UK ( Fox *et al.,* 2022). *O. sylvanus* shows a faint chequered pattern on both sides of the wings. Males can be differentiated from females by the presence of a conspicuous black sex brand on the forewing). The Large Skipper is univoltine in most of its range and overwinters as larva. In the UK, adults are active from the end of May to mid-August ( Newland & Tomlinson, 2010). In the southern areas it is bivoltine and it can be found as adult from end of April to October ( Vila *et al.*, 2018). *O. sylvanus* is common in rough grassland around woodland edges, forest clearings and urban parks. The main habitat requirement is the presence of uncut, tall grasses. Because of this, populations are negatively affected by intensive agriculture ( Newland & Tomlinson, 2010).

Caterpillars feed on a wide range of grasses ( Robinson *et al.*, 2010; Savela, 2022). They roll grass blades and use silken threads to build a shelter where they spend most of their lives, leaving only to feed. Females lay the dome-shaped, pearl-white eggs individually on the underside of grass blades.

The Large Skipper has a haploid chromosome number of 29 ( Federley, 1938 & Lorkovic, 1941), and its genome size has been estimated as 345 Mb using flow cytometry ( Mackintosh *et al*., 2019). Here we present a chromosome level assembly based on a female sample from Carrifran Wildwood, Scotland.

### Genome sequence report

**Figure 1.**
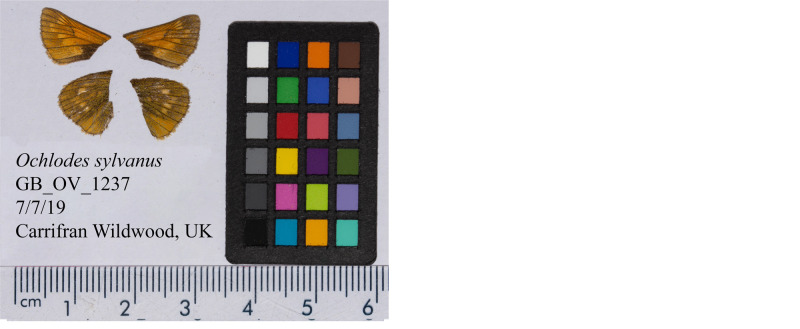
Photographs of forewings and hindwings of the *O. sylvanus female* specimen GB_OV_1237 (ilOchSylv3) used to generate Pacific BioSciences and Hi-C data. Dorsal (left) and ventral (right) surface views of wings from the specimen.

The genome was sequenced from one female *O. sylvanus* ( Figure 1) collected from Carrifan Wildwood, Scotland, UK (55.40, –3.34). A total of 23-fold coverage in Pacific Biosciences single-molecule HiFi long reads and 84-fold coverage in 10X Genomics read clouds were generated. Primary assembly contigs were scaffolded with chromosome conformation Hi-C data. Manual assembly curation corrected 101 missing joins and misjoins and removed 12 haplotypic duplications, reducing the assembly length by 1.06% and the scaffold number by 64.52%, and increasing the scaffold N50 by 8.56%.

**Figure 2.**
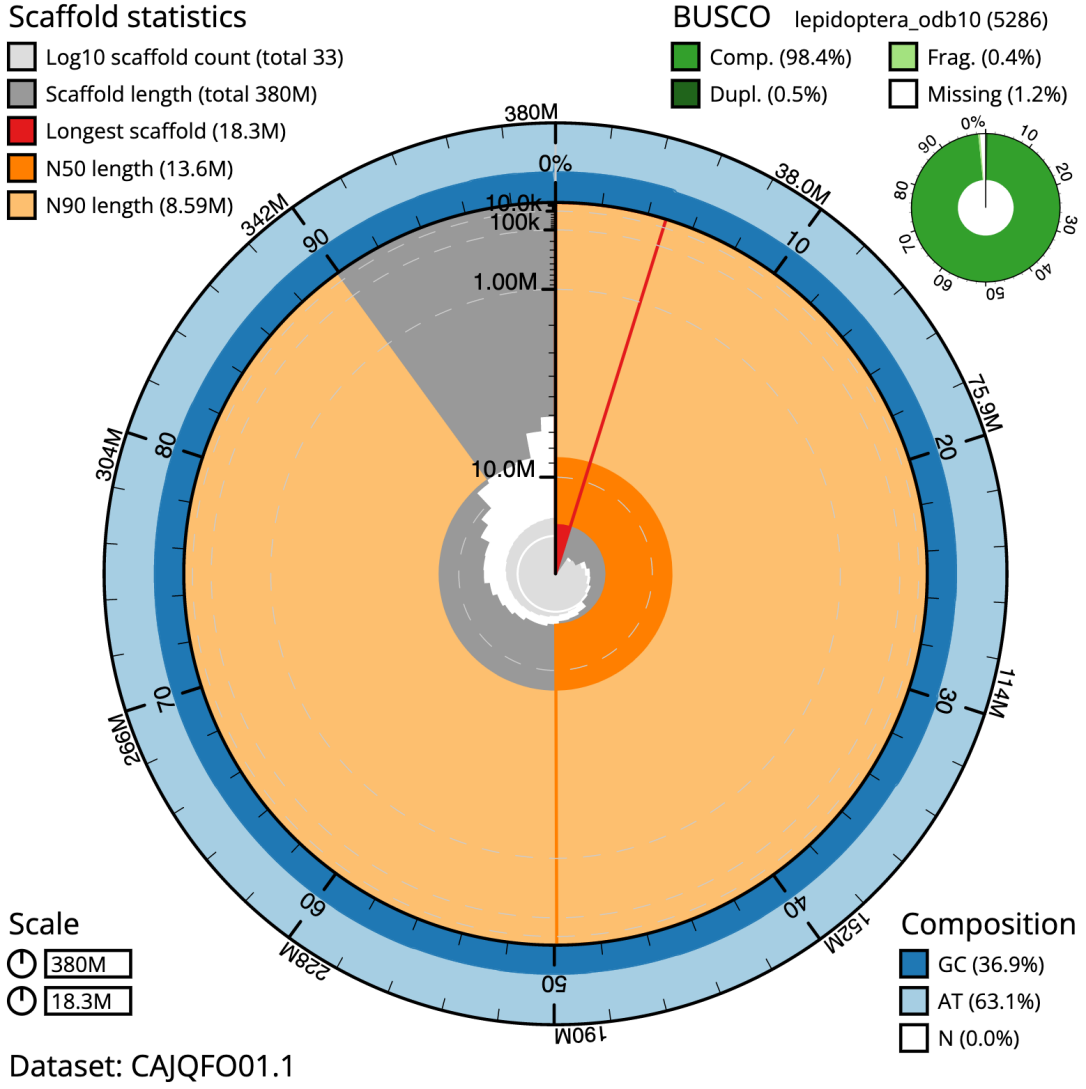
Genome assembly of *Ochlodes sylvanus*, ilOchSylv3.2: metrics. The BlobToolKit Snailplot shows N50 metrics and BUSCO gene completeness. The main plot is divided into 1,000 size-ordered bins around the circumference with each bin representing 0.1% of the 379,686,201 bp assembly. The distribution of scaffold lengths is shown in dark grey with the plot radius scaled to the longest scaffold present in the assembly (18,273,351 bp, shown in red). Orange and pale-orange arcs show the N50 and N90 scaffold lengths (13,557,832 and 8,588,389 bp), respectively. The pale grey spiral shows the cumulative scaffolds count on a log scale with white scale lines showing successive orders of magnitude. The blue and pale-blue area around the outside of the plot shows the distribution of GC, AT and N percentages in the same bins as the inner plot. A summary of complete, fragmented, duplicated and missing BUSCO genes in the lepidoptera_odb10 set is shown in the top right. An interactive version of this figure is available at https://blobtoolkit.genomehubs.org/view/ilOchSylv3.1/dataset/CAJQFO01.1/snail.

The final assembly has a total length of 380 Mb in 33 sequence scaffolds with a scaffold N50 of 14 Mb ( Table 1). Most (99.97%) of the assembly sequence was assigned to 30 chromosomal-level scaffolds, representing 28 autosomes and the W and Z sex chromosomes ( Figure 2– Figure 5; Table 2). Chromosome-scale scaffolds confirmed by the Hi-C data are named in order of size. While not fully phased, the assembly deposited is of one haplotype. Contigs corresponding to the second haplotype have also been deposited. The assembly has a BUSCO v5.3.2 ( Manni *et al.*, 2021) completeness of 98.4% (single 97.9%, duplicated 0.5%) using the lepidoptera_odb10 reference set.

**Table 1.** Genome data for *Ochlodes sylvanus*, ilOchSylv3.2.

Project accession data	
Assembly identifier	ilOchSylv3.2
Species	*Ochlodes sylvanus*
Specimen	ilOchSylv3
NCBI taxonomy ID	876063
BioProject	PRJEB43803
BioSample ID	SAMEA7523138
Isolate information	ilOchSylv3: female, whole organism (genome assembly); ilOchSylv4: female, whole organism (Hi- C); ilOchSylv5: male, whole organism (RNA-Seq)
Raw data accessions	
PacificBiosciences SEQUEL II	ERR6590586
10X Genomics Illumina	ERR6054646, ERR6054647, ERR6054648, ERR6054649
Hi-C Illumina	ERR6054650
PolyA RNA-Seq Illumina	ERR6363263
Genome assembly	
Assembly accession	GCA_905404295.2
*Accession of alternate* *haplotype*	GCA_905404205.1
Span (Mb)	380
Number of contigs	127
Contig N50 length (Mb)	6.8
Number of scaffolds	33
Scaffold N50 length (Mb)	13.6
Longest scaffold (Mb)	18.3
BUSCO *	C:98.4%[S:97.9%,D:0.5%], F:0.4%,M:1.2%,n:5,286
Genome annotation	
Number of protein- coding genes	13,451
Average length of coding sequence (bp)	1,475.95
Average number of exons per transcript	7.2
Average intron size (bp)	1,633.82

* BUSCO scores based on the lepidoptera_odb10 BUSCO set using v5.3.2. C = complete [S = single copy, D = duplicated], F = fragmented, M = missing, n = number of orthologues in comparison. A full set of BUSCO scores is available at https://blobtoolkit.genomehubs.org/view/ilOchSylv3.1/dataset/CAJQFO01.1/busco.

**Figure 3.**
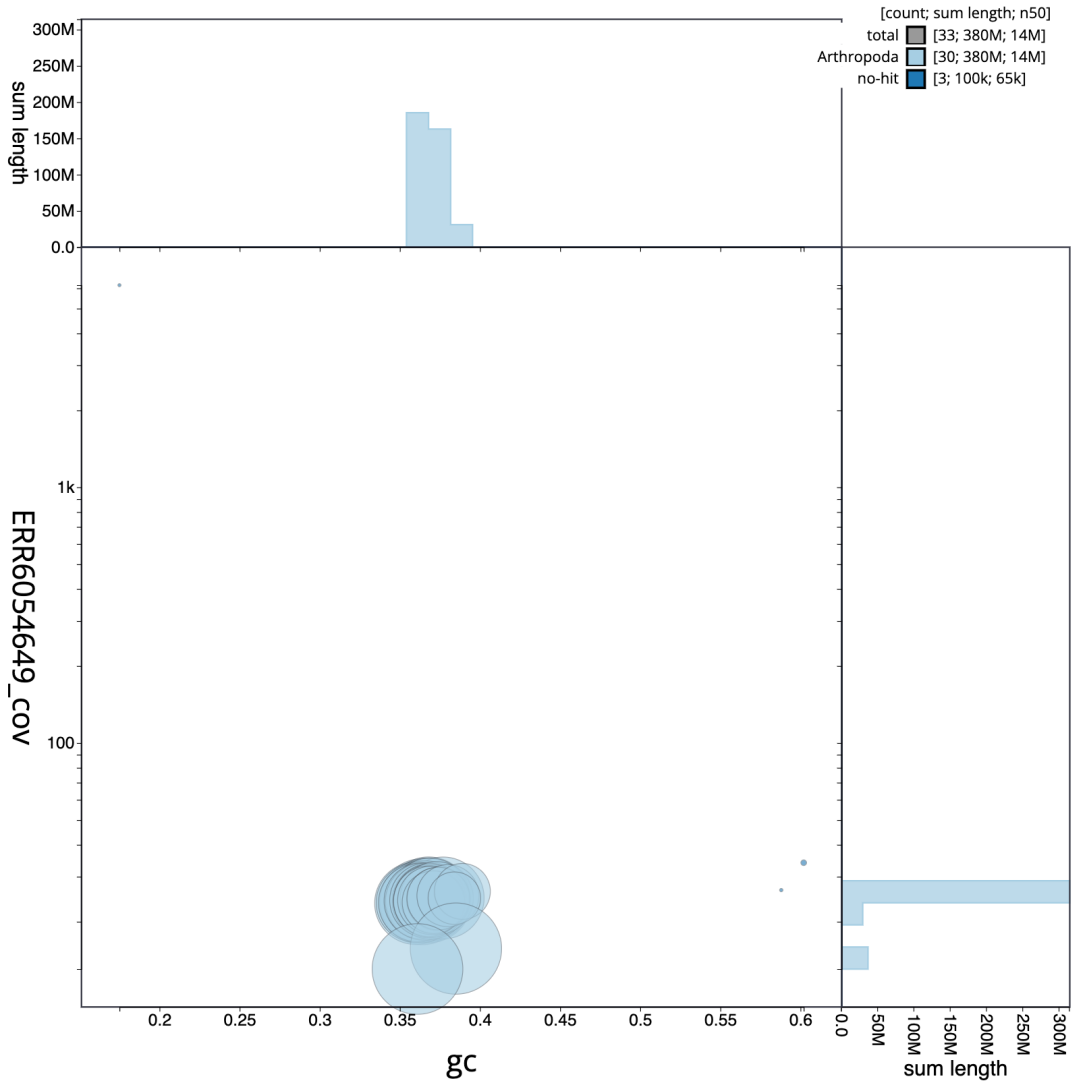
Genome assembly of *Ochlodes sylvanus*, ilOchSylv3.2: GC coverage. BlobToolKit GC-coverage plot. Scaffolds are coloured by phylum. Circles are sized in proportion to scaffold length. Histograms show the distribution of scaffold length sum along each axis. An interactive version of this figure is available at https://blobtoolkit.genomehubs.org/view/ilOchSylv3.1/dataset/CAJQFO01.1/blob.

**Figure 4.**
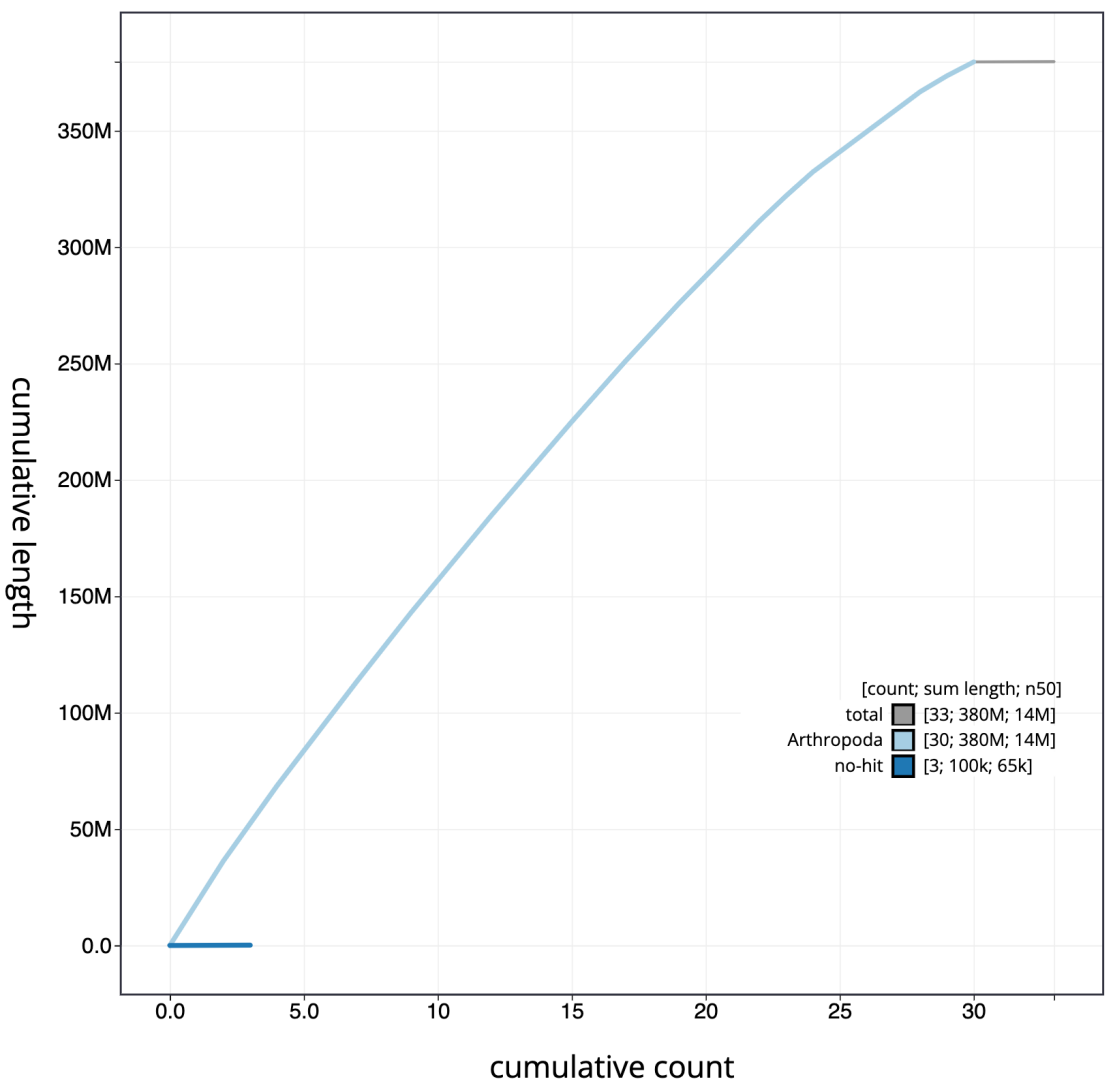
Genome assembly of *Ochlodes sylvanus*, ilOchSylv3.2: cumulative sequence. BlobToolKit cumulative sequence plot. The grey line shows cumulative length for all scaffolds. Coloured lines show cumulative lengths of scaffolds assigned to each phylum using the buscogenes taxrule. An interactive version of this figure is available at https://blobtoolkit.genomehubs.org/view/ilOchSylv3.1/dataset/CAJQFO01.1/cumulative.

**Figure 5.**
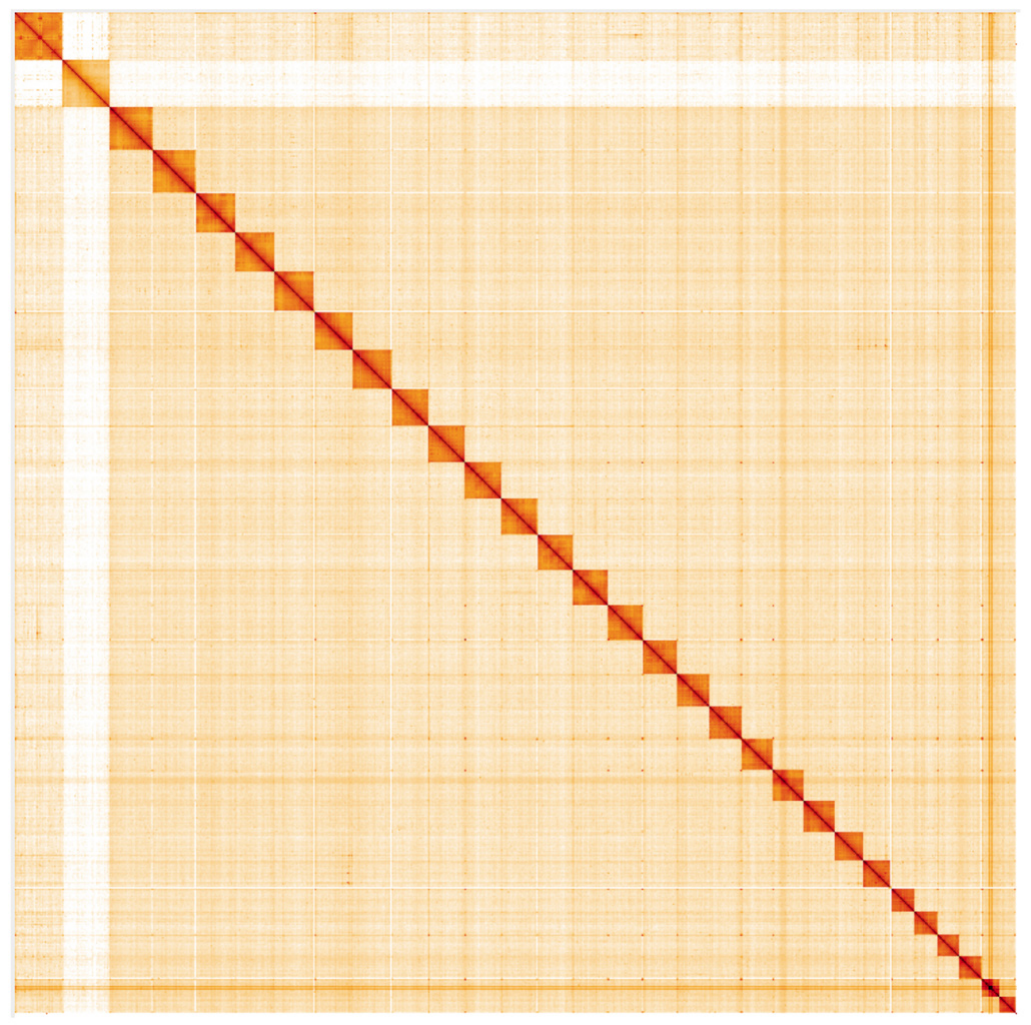
Genome assembly of *Ochlodes sylvanus*, ilOchSylv3.2: Hi-C contact map. Hi-C contact map of the ilOchSylv3.2 assembly, visualised using HiGlass. Chromosomes are shown in order of size from left to right and top to bottom. An interactive version of this figure may be viewed at https://genome-note-higlass.tol.sanger.ac.uk/l/?d=RYaBEd0ET66HAmyN3O4-qw.

**Table 2.** Chromosomal pseudomolecules in the genome assembly of *Ochlodes sylvanus*, ilOchSylv3.

INSDC accession	Chromosome	Size (Mb)	GC%
FR990124.1	1	16.21	36.4
FR990125.1	2	16.11	36.8
FR990126.1	3	15.22	36.8
FR990127.1	4	14.96	36.7
FR990128.1	5	14.9	36
FR990129.1	6	14.7	37.7
FR990130.1	7	14.59	36.3
FR990131.1	8	14.06	36.2
FR990132.1	9	13.88	36.6
FR990133.1	10	13.81	36
FR990134.1	11	13.56	36.6
FR990135.1	12	13.36	36.1
FR990136.1	13	13.36	36.9
FR990137.1	14	13.08	36.5
FR990138.1	15	12.83	36.4
FR990139.1	16	12.62	36.9
FR990140.1	17	12.25	36.7
FR990141.1	18	11.82	36.9
FR990142.1	19	11.78	36.9
FR990143.1	20	11.71	37.3
FR990144.1	21	10.92	36.8
FR990145.1	22	10.28	37
FR990146.1	23	8.82	37.4
FR990147.1	24	8.59	37.1
FR990148.1	25	8.58	37.4
FR990149.1	26	8.43	38
FR990150.1	27	6.83	38.9
FR990151.1	28	6.02	38.4
FR990122.1	W	18.27	38.5
FR990123.1	Z	18.04	36.1
FR990152.2	MT	0.02	17.1

### Genome annotation report

The ilOchSylv3.1 assembly was annotated using the Ensembl rapid annotation pipeline ( Table 1; GCA_905404295). The resulting annotation includes 28,177 transcribed mRNAs from 13,451 protein-coding and 3,418 non-coding genes. There are, on average, 7.20 exons and 6.20 introns per protein-coding transcript.

## Methods

### Sample acquisition and nucleic acid extraction

Two female *O. sylvanus* specimens (ilOchSylv3 and ilOchSylv4) were collected and identified by Konrad Lohse (University of Edinburgh). The specimens were caught using a net in Carrifran Wildwood, Scotland, UK (latitude 55.40, longitude –3.34). A male *O. sylvanus* specimen (ilOchSylv5) was collected by netting by Alex Hayward (University of Exeter) in Ruan Minor, Cornwall, UK (latitude 49.99, longitude –5.20). Both samples were preserved by freezing at –80°C.

DNA was extracted at the Tree of Life laboratory, Wellcome Sanger Institute. The ilOchSylv3 sample was weighed and dissected on dry ice. Whole body tissue was disrupted using a Nippi Powermasher fitted with a BioMasher pestle. High molecular weight (HMW) DNA was extracted using the Qiagen MagAttract HMW DNA extraction kit. Low molecular weight DNA was removed from a 20-ng aliquot of extracted DNA using 0.8X AMpure XP purification kit prior to 10X Chromium sequencing; a minimum of 50 ng DNA was submitted for 10X sequencing. HMW DNA was sheared into an average fragment size of 12–20 kb in a Megaruptor 3 system with speed setting 30. Sheared DNA was purified by solid-phase reversible immobilisation using AMPure PB beads with a 1.8X ratio of beads to sample to remove the shorter fragments and concentrate the DNA sample. The concentration of the sheared and purified DNA was assessed using a Nanodrop spectrophotometer and Qubit Fluorometer and Qubit dsDNA High Sensitivity Assay kit. Fragment size distribution was evaluated by running the sample on the FemtoPulse system.

RNA was extracted from whole organism tissue of ilOchSylv5 in the Tree of Life Laboratory at the WSI using TRIzol, according to the manufacturer’s instructions. RNA was then eluted in 50 μl RNAse-free water and its concentration was assessed using a Nanodrop spectrophotometer and Qubit Fluorometer using the Qubit RNA Broad-Range (BR) Assay kit. Analysis of the integrity of the RNA was done using Agilent RNA 6000 Pico Kit and Eukaryotic Total RNA assay.

### Sequencing

Pacific Biosciences HiFi circular consensus and 10X Genomics read cloud DNA sequencing libraries were constructed according to the manufacturers’ instructions. Poly(A) RNA-Seq libraries were constructed using the NEB Ultra II RNA Library Prep kit. DNA and RNA sequencing was performed by the Scientific Operations core at the WSI on Pacific Biosciences SEQUEL II (HiFi), Illumina HiSeq 4000 (RNA-Seq) and HiSeq X Ten (10X) instruments. Hi-C data were also generated from whole organism tissue of ilOchSylv4 using the Arima v2 kit and sequenced on the HiSeq X Ten instrument.

### Genome assembly

Assembly was carried out with Hifiasm ( Cheng *et al.*, 2021) and haplotypic duplication was identified and removed with purge_dups ( Guan *et al.*, 2020). One round of polishing was performed by aligning 10X Genomics read data to the assembly with Long Ranger ALIGN, calling variants with freebayes ( Garrison & Marth, 2012). The assembly was then scaffolded with Hi-C data ( Rao *et al.*, 2014) using SALSA2 ( Ghurye *et al.*, 2019). The assembly was checked for contamination and corrected using the gEVAL system ( Chow *et al.*, 2016) as described previously ( Howe *et al.*, 2021). Manual curation was performed using gEVAL, HiGlass ( Kerpedjiev *et al.*, 2018) and Pretext ( Harry, 2022). The mitochondrial genome was assembled using MitoHiFi ( Uliano-Silva *et al.*, 2021), which performed annotation using MitoFinder ( Allio *et al.*, 2020). The genome was analysed and BUSCO scores generated within the BlobToolKit environment ( Challis *et al.*, 2020). Table 3 contains a list of all software tool versions used, where appropriate.

**Table 3.** Software tools and versions used.

Software tool	Version	Source
BlobToolKit	3.2.9	Challis *et al.*, 2020
freebayes	1.3.1-17-gaa2ace8	Garrison & Marth, 2012
gEVAL	N/A	Chow *et al.*, 2016
Hifiasm	0.12	Cheng *et al.*, 2021
HiGlass	1.11.6	Kerpedjiev *et al.*, 2018
Long Ranger ALIGN	2.2.2	https:// support.10xgenomics. com/genome-exome/ software/pipelines/latest/ advanced/other-pipelines
MitoHiFi	1	Uliano-Silva *et al.*, 2021
PretextView	0.2	Harry, 2022
purge_dups	1.2.3	Guan *et al.*, 2020
SALSA	2.2	Ghurye *et al.*, 2019

### Genome annotation

The Ensembl gene annotation system ( Aken *et al.*, 2016) was used to generate annotation for the *O. sylvanus* assembly (GCA_905404295.1). Annotation was created primarily through alignment of transcriptomic data to the genome, with gap filling via protein to-genome alignments of a select set of proteins from UniProt ( UniProt Consortium, 2019).

## Data Availability

European Nucleotide Archive: *Ochlodes sylvanus* (Large Skipper). Accession number PRJEB43803; https://identifiers.org/ena.embl/PRJEB43803 ( Wellcome Sanger Institute, 2022) The genome sequence is released openly for reuse. The *Ochlodes sylvanus* genome sequencing initiative is part of the Darwin Tree of Life (DToL) project. All raw sequence data and the assembly have been deposited in INSDC databases. Raw data and assembly accession identifiers are reported in Table 1.

## Author information

Members of the Wellcome Sanger Institute Tree of Life programme are listed here: https://doi.org/10.5281/zenodo.4783585.

Members of Wellcome Sanger Institute Scientific Operations: DNA Pipelines collective are listed here: https://doi.org/10.5281/zenodo.4790455.

Members of the Tree of Life Core Informatics collective are listed here: https://doi.org/10.5281/zenodo.5013541.

Members of the Darwin Tree of Life Consortium are listed here: https://doi.org/10.5281/zenodo.4783558.
