# Supplementary material for: The genome sequence of the Large Skipper, Ochlodes sylvanus, (Esper, 1777)
Source: Wellcome Open Res. 2023 Feb 14;8:75. [Version 1] doi: 10.12688/wellcomeopenres.18788.1 (PMC10435925; doi:10.12688/wellcomeopenres.18788.1)
Supplement: Peer review history

# Peer review: Reviewer response for version 1

**DOI:** 10.21956/wellcomeopenres.20833.r63848

**Published:** 2023-08-17

**Authors:** Li-Wei Wu

**Affiliations:** 1 Tunghai University, Taichung, China

The report presents the genomic sequences of *Ochlodes sylvanus*, with a genome size of 380 megabases (99.97% assembly). The species has a total of 32 pseudomolecules (including W and Z sex chromosomes) and a mitochondrial genome of 17.1 kilobases. This is a standard report to describe a genome data, characterized by its comprehensible analysis and writing style. It serves as an excellent reference for subsequent researchers. I only have minor suggestions regarding the readability of the content while reading:

1. The “Scaffold statistics” and “Scale” used in Figure 2 are not entirely clear. For instance, the light gray color indicates scaffold count, while the dark gray color represents length. It's not evident how this figure effectively portrays the count and length of scaffolds. Additionally, in the lower-left section of Figure 2, there are two scales mentioned, 380M and 18.3M. It's unclear where these scales are positioned. I suggest to make slight modifications to the presentation of Figure 2 to enhance reader comprehension.
2. In Figure 3, the labeling "Scaffolds are coloured by phylum" might be redundant. In addition, Figure 4 seems somewhat unnecessary, as the accompanying text already indicates that the genome is 380M in size. Perhaps it could be merged with Figure 3 or just removed it out for concising the reading.

Are sufficient details of methods and materials provided to allow replication by others?

Yes

Is the rationale for creating the dataset(s) clearly described?

Yes

Are the datasets clearly presented in a useable and accessible format?

Yes

Are the protocols appropriate and is the work technically sound?

Yes

Reviewer Expertise:

Museomics, insect diversity

I confirm that I have read this submission and believe that I have an appropriate level of expertise to confirm that it is of an acceptable scientific standard.

# Peer review: Reviewer response for version 1

**DOI:** 10.21956/wellcomeopenres.20833.r60336

**Published:** 2023-08-17

**Authors:** Noel Vella

**Affiliations:** 1 Università ta' Malta, Msida, Malta

The article presents a high-quality genome for a female specimen of *Ochlodes Sylvanus* (Lepidoptera: Hesperiidae) collected from Scotland. The genome is reported to be 380 Mb and has been scaffolded into 30 chromosomes, including the W and Z sex chromosomes. The mtDNA genome was also assembled into 17.1 kb. Genes were annotated on Ensembl which identified 13451 protein coding genes. Protocols used are clearly outlined and all data is publicly available.

 Minor typo: remove close brackets from behind the term forewing (second paragraph).

Are sufficient details of methods and materials provided to allow replication by others?

Yes

Is the rationale for creating the dataset(s) clearly described?

Yes

Are the datasets clearly presented in a useable and accessible format?

Yes

Are the protocols appropriate and is the work technically sound?

Yes

Reviewer Expertise:

Genetics and genomics

I confirm that I have read this submission and believe that I have an appropriate level of expertise to confirm that it is of an acceptable scientific standard.

# Peer review: Reviewer response for version 1

**DOI:** 10.21956/wellcomeopenres.20833.r58328

**Published:** 2023-06-12

**Authors:** Weibin Jiang

**Affiliations:** 1 Shanghai Normal University, Shanghai, Shanghai, China

The authors here present a high-quality genome of an individual female from the species *Ochlodes sylvanus* , from the subfamily Hesperiinae. The reported genome is 380 Mb in length has a high contiguity and level of completeness. Gene annotation of this assembly on Ensembl identified 13,451 protein coding genes. The datasets presented are clear and freely accessible online.

 I have minor comments on specific parts of the article:

- The abstract repotted that the mitochondrial genome has also been assembled and is 17.1 kilobases in length. However, I can not find them in the text.
- Please delete the extra parentheses in the  background section " *Males can be differentiated from females by the presence of a conspicuous black sex brand on the forewing).*"

Are sufficient details of methods and materials provided to allow replication by others?

Yes

Is the rationale for creating the dataset(s) clearly described?

Yes

Are the datasets clearly presented in a useable and accessible format?

Yes

Are the protocols appropriate and is the work technically sound?

Yes

Reviewer Expertise:

phylogeny of Lepidoptera

I confirm that I have read this submission and believe that I have an appropriate level of expertise to confirm that it is of an acceptable scientific standard.
